# High variability of genomic instability and gene expression profiling in different HeLa clones

**DOI:** 10.1038/srep15377

**Published:** 2015-10-20

**Authors:** Annalisa Frattini, Marco Fabbri, Roberto Valli, Elena De Paoli, Giuseppe Montalbano, Laura Gribaldo, Francesco Pasquali, Emanuela Maserati

**Affiliations:** 1 Institute of Genetic and Biomedical Research (IRGB), National Research Council (CNR), via Fantoli 15/16, 20090 Milan, Italy; 2 Department of Clinical and Experimental Medicine (DMCS), University of Insubria, via J.H. Dunant 5, 21100 Varese, Italy; 3 Institute for Health and Consumer Protection, European Commission, I Via Enrico Fermi 2749, 21027 Ispra (VA), Italy

## Abstract

The HeLa cell line is one of the most popular cell lines in biomedical research, despite its well-known chromosomal instability. We compared the genomic and transcriptomic profiles of 4 different HeLa batches and showed that the gain and loss of genomic material varies widely between batches, drastically affecting basal gene expression. Moreover, different pathways were activated in response to a hypoxic stimulus. Our study emphasizes the large genomic and transcriptomic variability among different batches, to the point that the same experiment performed with different batches can lead to distinct conclusions and irreproducible results. The HeLa cell line is thought to be a unique cell line but it is clear that substantial differences between the primary tumour and the human genome exist and that an indeterminate number of HeLa cell lines may exist, each with a unique genomic profile.

Since HeLa was first established as a human cancer cell line in 1952^1^, it has become probably the most-used human cell line in biological research. The cell line was established from the invasive cervical adenocarcinoma of a young patient, Henrietta Lacks, who eventually died in 1951, and it was the first successful attempt to establish a culture of immortalized human cells. Starting with the pioneering research of Jonas Salk, who developed the first polio vaccine^2^, the use of HeLa cells has contributed to many fundamental scientific breakthroughs.

HeLa cells have been employed to investigate cancer, AIDS mechanisms^3^, and the effects of drugs^4^, toxins^5^ and radiation^6^. These cells have also been used in the Nobel-winning experiments that led to the discovery of telomeric activity^7^,^8^.

Furthermore, to verify the gene-editing effects involved in specific cellular processes, gene expression profiles and proteome analyses have been applied to HeLa cells^9^,^10^,^11^. The widespread use of this cell line is mainly due to the ease with which they can be handled and manipulated in different conditions.

Starting from the 1950 s, approximately 80,000 studies have reported results obtained using the HeLa cell line as a physiological model. In the majority of these studies, the source of the HeLa cell line is not specified, implying a lab-to-lab origin (Supplementary Table S1).

Several published studies reported that HeLa cells are characterized by extensive chromosome instability (CIN)^12^,^13^,^14^,^15^,^16^,^17^,^18^,^19^ (Supplementary Table S1), although one study concluded that they are stable and able to maintain a constant number of chromosomes mitosis after mitosis^20^.

A few years after the establishment of the line, one of the first studies on the metaphase spreads of clonal cell strains of HeLa was published in 1958^21^, showed a relatively narrow chromosome number distribution between 75 and 82. The large number of duplications and deletions compared with the normal human genome has already been extensively described^19^ (Supplementary Table S1), and as chromosomes provide genome identity, it might even be argued that the HeLa genome is no longer a human one^22^. In 1991, it was even proposed that the HeLa cell could be considered a new species (*Helacyton gartleri*)^23^.

To our knowledge, all of the reports regarding HeLa CIN have been conducted on a single cell clone or on sub-clones^20^,^24^. Recently, the HeLa genome and transcriptome was exhaustively characterized by deep RNA and DNA sequencing performed on a single clone from CLS Cell Lines Service GmbH (the HeLa Kyoto cell line)^25^. To investigate the reported instability of the HeLa cell lines and to verify the possibility that erroneous conclusions could be generated by the use of HeLa cells obtained from different laboratories, we compared different “batches” obtained from four Italian laboratories (HeLaSR, HeLaV, HeLaP, and HeLaH). HeLaP and HeLaH were derived from the same batch but had been cultured in two different laboratories for approximately 8 years; similarly, HeLaV was derived from the same batch as HeLaSR but had been cultured in two different laboratories for approximately 12 years.

## Results

### HeLa karyotyping and a-CGH show genomic instability

We evaluated the genomic stability of these “batches” by karyotyping, array-comparative genomic hybridization (a-CGH) and fluorescent *in situ* hybridization (FISH described in the next section).

Cytogenetic analysis was performed on 20 metaphase spreads stained using the quinacrine-based Q-banding technique (QFQ). The HeLa cells were near-triploid, containing a range of 60–66 chromosomes in HeLaSR, 56–79 in HeLaV, 71–79 in HeLaP, and 63–79 in HeLaH. In addition, approximately 10% of the metaphase spreads of clone HeLaSR exhibited chromosome numbers ranging from 101 to 148, whereas HeLaV (derived from the HeLaSR batch) exhibited a range of 124–198 chromosomes in approximately 30% of its metaphase spreads. We excluded the coexistence of two different sub-clones by monoclonal sub-culturing.

Ten HeLaV clones, derived from a monoclonal sub-culture, showed the presence of both metaphase populations (with a range of 49–81 and a range of 92–240 chromosomes), suggesting that a single cell could carry the inherent defect, possibly generated by new mutation events. For our FISH and transcriptomic analysis, we used the batch HeLaSR because it showed fewer mitoses with double the modal chromosome number compared to HeLaV.

**Figure 1:**
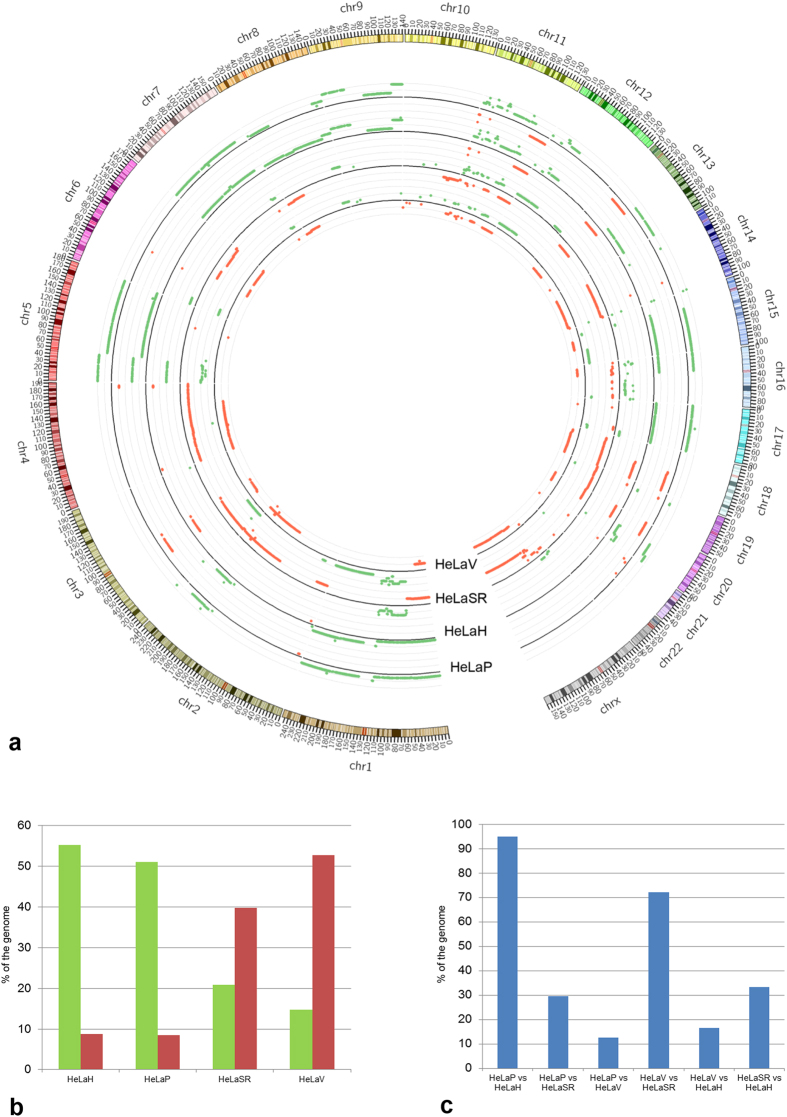
The genomic landscape of the 4 HeLa cell lines. (**a**) Circos plot of the four HeLa genomes compared with the diploid human genome, with tracks representing the gain (green) and loss (red) of genomic material. (**b**) The histogram summarizes the percentage of the gain (green) and loss (red) of genomic material in the 4 cell lines compared to the diploid human genome, highlighting the separate evolution of the two pairs of HeLa clones (HeLaH and HeLP vs HeLaSR and HeLaV). (**c**) The histogram shows the percentage of similarity in genomic content in each HeLa clone compared with the other lines and emphasizes that the two more similar lines, based on their common origin (HeLaH and HeLaP; HeLaSR and HeLaV), bear differences in their genomic content.

To characterize all of the genomic imbalances of the four “batches”, we performed a-CGH. As showed in the Circos plot^26^ (Fig. 1A), a comparison between the HeLa DNA content and the diploid human genome evinced a substantial difference among the four lines analysed. The comparison of the 4 HeLa clones underscored that although the amount of gain and loss of genomic material compared to the human diploid genome is highly variant among the 4 clones, there is similarity within the pairs with a common origin, i.e., HeLaH/HeLaP vs HeLaSR/HeLaV (Fig. 1B).

To express the similarities among the HeLa clones, we calculated the percentage of the genomes with the same annotation (diploid, deleted or amplified). The results, depicted in Fig. 1C, show high similarity within the two pairs HeLaSR/HeLaV and HeLaH/HeLaP. We emphasize that even recently split-out batches (HeLaSR vs HeLaV and HeLaH vs HeLaP) present specific gains or losses and that these new events are acquired and stably maintained. A possible hypothesis to explain the different chromosome imbalances between the two pairs could be ancestral mutational events that conferred an evolutionary advantage in DNA losses or gains.

### FISH analysis highlights new specific markers

To identify chromosomal rearrangements and to verify the presence of HeLa-specific markers observed by karyotyping^17^,^20^, we performed a FISH analysis to paint chromosomes 1, 3, 5, 9, 13 and 19 in HeLaSR and HeLaP because they appeared to be the two most different clones by a-CGH. As shown in Table 1, HeLaP displays almost all of the HeLa-specific markers previously identified^12^,^20^,^25^, whereas HeLaSR has lost most of them. In addition, we found in each clone the presence of new specific markers (Supplementary Fig. S1), confirming an independent evolution of each batches.

### Transcriptomic analysis reveals that different batches exposed to hypoxic stimulus display differences in gene expression

Our genomic analysis uncovered deep variability between thebatches , and because genomic gains or losses or whole-chromosome aneuploidy can have drastic consequences on gene expression^27^, we performed a whole-transcriptome analysis to verify the transcriptional effects of this differential chromosome instability. The microarray expression analysis was performed on HeLaSR and HeLaP, the batches most different in terms of genomic content. These cell lines were exposed to hypoxic conditions, chosen because the induced hypoxic pathway is well characterized^28^,^29^.

**Figure 2:**
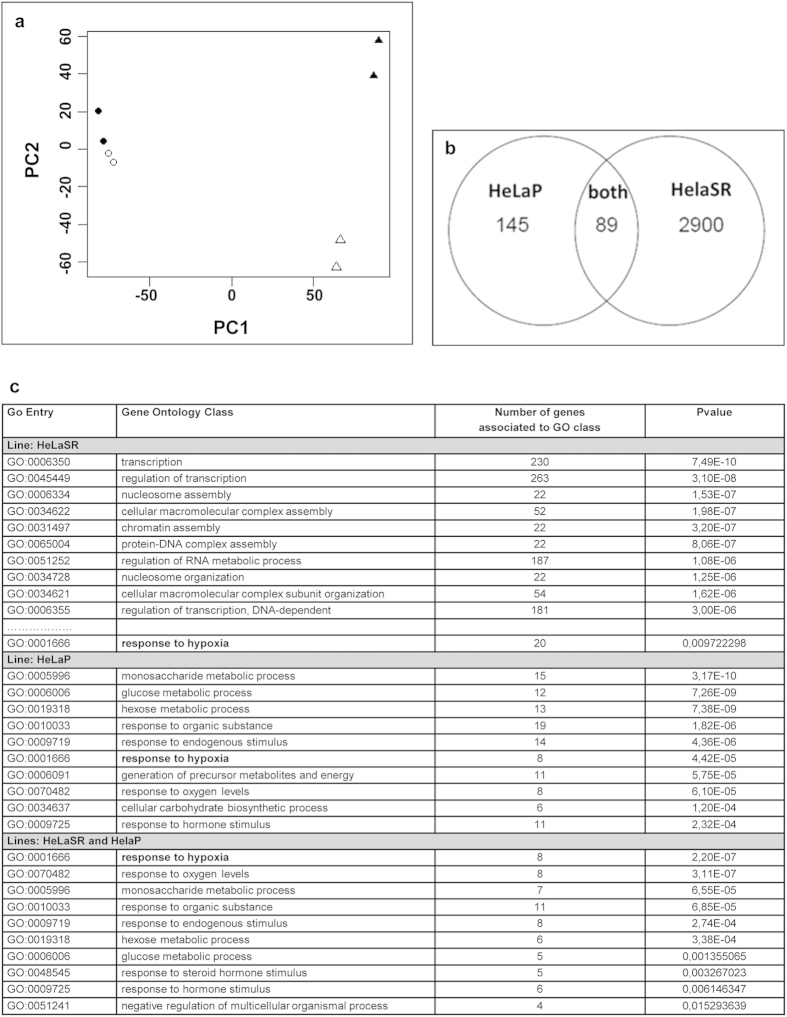
Transcriptional landscape. (**a**) Principal component analysis (PCA) of the transcriptome. The PCA maps the entire dataset on a two-axis graph (principal components, PC1 and PC2), where the distances account for similarity. Untreated HeLaP cells (white circles) and HeLaP cells after hypoxia (black circles) are closer to each other than untreated HeLaSR cells (white triangles) are to hypoxic HeLaSR cells (black triangles), suggesting that hypoxia exerts a stronger effect on HeLaSR cells than on HeLaP cells. (**b**) Venn diagram showing the number of genes either upregulated or downregulated due to hypoxia in the HeLaP and HeLaSR clones compared to their respective controls. (**c**) Gene ontology of genes either upregulated or downregulated by hypoxia in HeLaSR and HeLaP cells.

A principal-component analysis (PCA) was performed to determine the expression trends within the dataset. PCA is a useful technique to reduce the dimensionality of large data sets and to visually assess similarities and differences between samples^30^. PCA was used to identify trends in the regulation of genes induced by hypoxic exposure and to map the entire dataset on a two-axis graph (principal components, PC1 and PC2) where the distances account for similarity (Fig. 2A); the closer the distance between samples, the more they are similar. The main divergence was due to two different clones, HeLaSR vs HeLaP (triangles and circles, respectively), as demonstrated by the first principal component (PC1). The second component (PC2), summarizing the effects of hypoxia, highlighted strong changes in HeLaSR and mild changes in HeLaP. Indeed, untreated HeLaP and HeLaP after hypoxia are closer to each other than untreated HeLaSR is to HeLaSR after hypoxia, meaning that hypoxia has a stronger effect in HeLaSR than in HeLaP (Fig. 2A). Furthermore, we identified the genes regulated by hypoxic treatment in these two clones: 2,900 (1,666 genes upregulated and 1,234 downregulated) genes were mis-regulated only in HeLaSR, whereas 145 genes were mis-regulated only in HeLaP (104 up and 41 down). A total of 89 genes (88 up and 1 down) were mis-regulated in both cell lines (Fig. 2B).

These three gene lists were analysed for gene ontology (GO) enrichment (Fig. 2C). The more significant GO classes those that are differentially expressed between HeLaSR and HeLaP, demonstrating the large differences in the expression profiles of the two cell lines in response to hypoxia.

The classes related to hypoxia were present in both, but they were not the most significant (Fig. 2C). On the contrary, when we analysed the 89 mis-regulated genes common to both cell lines, the most enriched classes are related to hypoxia (Fig. 2B,C). These results suggest that the different genomic contents of the two clones have a remarkable influence on basal gene expression and, consequently, on the response to a hypoxic stimulus. The cells activate different pathways, but interestingly, the regulation of hypoxia-related genes is preserved.

### Real-time RT-PCR confirmed the microarray data

A subset of regulated genes identified for their relevance in the hypoxic response were validated by real-time RT-PCR (qPCR) (Supplementary Table S2). The trend of the array data was confirmed by qRT-PCR (Supplementary Table S3).

### Copy number variation influences gene expression

**Figure 3:**
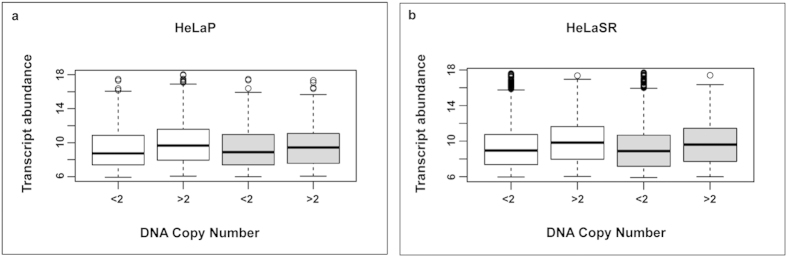
Gene expression by copy number in HeLaP and HeLaSR cells. Transcript abundance (arbitrary unit) is positively correlated with gene copy number. (**a**) HeLaP gene expression in untreated (white) and hypoxic (grey) cells. (**b**) HeLaSR gene expression in untreated (white) and hypoxic (grey) cells. A general lack of dosage compensation was observed in both cell lines.

Because significant chromosome copy number alterations are frequently associated with gene expression changes in the affected regions^31^, we evaluated the possible effects of copy number variation on gene expression^32^ in our samples. Based on the data obtained with a-CGH and the expression array performed on HeLaP and HeLaSR samples as previously described, a general lack of dosage compensation was observed in HeLaP (Fig. 3A) and in HeLaSR (Fig. 3B), meaning that gene dosage is correlated to gene expression.

## Discussion

Genetic aberrations, such as gene amplification, deletions, and loss of heterozygosity, are hallmarks of cancer and are major contributors to the neoplastic process through the accumulation of mutations in specific genes. The mechanisms through which these mutations are generated are the subject of continued debate^33^,^34^,^35^,^36^,^37^, and several cancer-predisposing mutations affect genes that are responsible for maintaining the integrity and number of chromosomes during cell division.

The main factors that control chromosome stability are telomere maintenance, cell division mechanisms, and the mitotic checkpoint that ensures correct chromosome segregation^38^,^39^,^40^.

Consequently, the archetypical transformation of tumour cells results in CIN^41^,^42^. Established cell lines have been traditionally used to characterize the biological significance of specific genomic aberrations identified in primary tumours^43^ and to test the therapeutic efficacy of anticancer agents^44^.

HeLa was the first cultured cancer line^45^. Although its CIN has already been extensively investigated^19^ (see Supplementary Table S1), demonstrating the low degree of similarity with the initial tumour and with the human diploid genome, this cell line is largely used in biomedical research. The HeLa cell line is among the most frequently utilized models in research to validate drugs for cancer therapy^46^ and for genetic/epigenetic manipulation using demethylation agents^47^, siRNA and expression vectors to study gene function^48^,^49^,^50^.

Here, we report the high level of CIN in HeLa clones obtained from different laboratories. We demonstrated that each clone accumulates genomic variability in a time-dependent manner. We speculate that some chromosomal rearrangements or point mutations that randomly arose in each clone increased survival, leading to a kind of “genetic drift” in clonal variants.

This conclusion is evident in the differing DNA content of the clones HeLaSR and HeLaV, which showed a loss of genomic DNA, whereas HeLaH and HeLaP exhibited a gain of genomic DNA in comparison with the diploid human genome. We speculate that even slightly different culture conditions may produce this divergence. The number of passages that a cell line undergoes can certainly lead to extensive modifications in growth rate, morphology, aneuploidy, chromosome alterations, gene expression and drug sensitivity, depending on the culture environment^51^,^52^,^53^,^54^,^55^. In addition, we demonstrated that the genomic gains or losses or whole-chromosome aneuploidy is specific to each analysed clone (Fig. 1A) and that these differences in genomic content have a remarkable influence on basal gene expression (Fig. 2A). We showed that in response to a hypoxic stimulus, each cell line activates different pathways, although the regulation of genes related to hypoxia is conserved (Fig. 2A,C).

These results suggest that different HeLa batches used for the same experiment may yield different results. The large differences in the basal gene expression profiles suggest that the use of uncharacterized clones may lead to faulty conclusions and to irreproducible results in studies of gene function and pathway analysis. With respect to genomic content, the use of uncharacterized batches of HeLa cells^56^ may result in different behaviours between clones in response to specific stimuli, such as cancer drugs, used in biomedical research.

Our results suggest that not a single HeLa cell line, or even a set of similar HeLa cell lines, exists. Rather, an indeterminate number of clones exist, each carrying large genomic differences that lead to different expression profiles. The HeLa cell recalls the main character of the novel by Pirandello (the Italian poet awarded the 1934 Nobel Prize in Literature): “One, no one, one hundred thousand”^57^: the HeLa cell line is thought to be a unique cell line (“one”), but it is clear that large differences exist between the tumour from which it was derived and the human genome (“no one”) and that an indeterminate number of HeLa cell lines are scattered in laboratories worldwide (“one hundred thousand” or more), each with a unique genomic profile.

## Methods

### Cell lines

The cell lines HeLaP, HeLaH, HeLaSR and HeLaV were obtained from four Italian laboratories. HeLaP and HeLaH were derived from the same cell-line batch but were cultured in two different laboratories for approximately 8 years. Similarly, HeLaSR and HeLaV were derived from the same cell-line batch but were cultured in two different laboratories for approximately 12 years. In our lab, the mycoplasma-free cell lines were cultured in DMEM-F12 (Euroclone SpA, Milan, Italy) with 10% heat-inactivated foetal bovine serum (Euroclone), 1 mM glutamine, 100 U/ml penicillin and 100 μg/ml streptomycin and were incubated at 37 °C and 5% CO_2_. The cells were cultured to reach a confluence of 80–90%.

### Cytogenetic analysis

Metaphase spreads were obtained from the four cell lines. After culturing, the cells were treated with 0.04 μg/ml colcemid for 2 hours. The cells were harvested by treatment with a 1× trypsin/EDTA solution for approximately 5 min. A hypotonic treatment was performed with 0.96% Na citrate for 15 min at 37 °C, and the cells were subsequently fixed in a fresh methanol/acetic acid (3/1, v/v) solution. Metaphase spreads were stained overnight with 0.005% quinacrine mustard solution in McIlvaine buffer (QFQ-banding), and standard cytogenetic analyses were performed with a Leica D5000B fluorescent microscope.

### Monoclonal subculturing by limiting dilution

To exclude the possibility that the HeLaV and HeLaSR cell lines arose from 2 different HeLa subclones, we obtained a cell culture derived from a single cell by limiting-dilution culture. We prepared serial dilutions to obtain a suspension containing 1 cell/μl. A single μl of the final cell suspension was seeded into each well of a 96-well plate containing growth medium. The presence of a single cell was evaluated by microscopy, and the cells were incubated at 37 °C and 5% CO_2_ overnight. After one day, we used microscopy to confirm the presence of a single cluster of cells (2 or more) per well, implying a single-cell origin, and we excluded any wells with double or multiple clusters of cells. The monoclonal cell lines were trypsinised and seeded in T25 flasks to obtain sufficient cells for the metaphase spread analysis.

### Fluorescence *in situ* hybridization experiments

Metaphase spreads were hybridized with Cytocell Aquarius Whole Chromosome Painting probes (WCP Probes) using the codenaturation protocol. Briefly, 20 μl of ready-to-use probes were deposited on the slide and sealed with a coverslip using rubber cement. Codenaturation was performed on a HyChrome hybridization machine at 75 °C for 2 min, and hybridization was conducted overnight. The slides were then washed for 2 min in 0.4× SSC buffer at 72 °C and for 30 min in 2× SSC/0.05% Tween 20 at room temperature. Metaphases spreads were then counterstained in 2× SSC buffer with 200 ng/ml of 4,6-diamidino-2-phenylindole (DAPI) and then mounted with an antifade solution (Vector Laboratories INC., Burlingame, CA, USA). The samples were analysed with a Leica DM5000B fluorescence microscope, and images were captured with the Leica QFISH software system.

### DNA extraction

DNA was extracted from approximately 10 × 10^7^ cells using the DNeasy Blood and Tissue kit (Qiagen S.r.l. Milan, Italy) according to the manufacturer’s protocol. Genomic DNA was quantified using an ND-1000 UV-Vis spectrophotometer (Thermo Scientific, Wilmington, DE, USA).

### a-CGH analysis

a-CGH was performed on an Agilent microarray platform (Agilent Technologies Inc., Santa Clara, CA USA) with an Agilent’s human 4 × 180 K CGH slide. Sample preparation, labelling, and microarray hybridization were performed according to the Agilent CGH Enzymatic Protocol version 7.3. Slides were scanned using the Agilent G2565CA scanner and analysed using the Agilent Feature Extraction 10.7.3.1 software. The a-CGH profile was extrapolated using the Agilent Genomic Workbench 6.5.0.18 software with the following parameters: ADM-2 threshold 6, Fuzzy Zero ON, and Centralization OFF. Coverage plots were generated using the Circos visualization tool compared with the 2.2 × 10^9^ genome covered by the Agilent probes.

### Hypoxic treatment

Before administering the hypoxia treatment, the HeLaP and HeLaSR cells were maintained at 70–80% confluence. The medium was refreshed before the hypoxia treatment. Cells (two flasks per cell line) were maintained under hypoxic (1% O_2_, 5% CO_2_, 94% N_2_) conditions or normoxic (95% air and 5% CO_2_) conditions for 24 h.

### RNA extraction

Total RNA was purified from the HeLa cells using the RNeasy Plus kit (Qiagen). RNA was quantified using an ND-1000 UV-Vis spectrophotometer (Thermo Scientific, Wilmington, DE, USA), and the integrity of the RNA was assessed with the Agilent 2100 Bioanalyzer (Agilent Technologies Inc.) according to the manufacturer’s instructions. All of the RNA samples used in this study exhibited a 260/280 ratio above 1.9 and an RNA Integrity Number (RIN) above 9.0.

### Microarray expression profiling

The microarray experiment included two biological replicates per treatment. All sample-labelling, hybridization, washing, and scanning steps were conducted according to the manufacturer’s specifications. Briefly, Cy3-labelled cRNA was generated from 50 ng input total RNA using the One Color Quick Amp Labelling Kit (Agilent Technologies Inc.). For every sample, 600 ng cRNA from each labelling reaction (with a specific activity above 9.0) was hybridized using the Gene Expression Hybridization Kit (Agilent Technologies Inc.) to the Agilent Whole Human Genome Oligo Microarray (Agilent Technologies Inc.), which is in a 4 × 44k 60-mer slide format, where each of the 4 arrays represents approximately 41,000 unique genes and transcripts. After hybridization, the slides were washed and then scanned with the Agilent G2565BA Microarray Scanner (Agilent Technologies Inc.). The fluorescence intensities of the scanned images were extracted and pre-processed using the Agilent Feature Extraction Software (10.7.3.1).

### Gene expression data analysis

Quality control and array normalization were performed in the R statistical environment using the Agi4 × 44PreProcess (v 1.18.0) package, which was downloaded from the Bioconductor web site. The normalization and filtering steps were based on those described in the Agi4 × 44PreProcess reference manual. Briefly, the Agi4 × 44PreProcess options were set to use the Mean Signal and the BG Median Signal as foreground and background signals, respectively. The data were normalized between arrays using the quantile method. Genes with a fold change greater than 1 log_2_ were designated as modulated. All of the above computations were conducted using the R statistical programming environment. Principal-component analysis (PCA) was performed on all genes under investigation to determine their expression trends within the dataset. PCA is a useful technique to reduce the dimensionality of large data sets. The expression analysis systematic explorer (EASE) biological theme analysis of the regulated genes was conducted online using DAVID.

### Quantitative real-time PCR validation of microarray data

A real-time quantitative PCR (qRT-PCR) analysis was performed, in triplicate, on the same RNA samples that were used for the microarray hybridization to validate the microarray results. One μg of total RNA was retro-transcribed using SuperScript II (Life Technologies, Carlsbad, CA, USA). qRT-PCR was performed using the iQ SYBR® Green supermix (Bio-Rad, Hercules, CA, USA,) on the ABI Prism 7000 platform (Applied Biosystems) with the following thermal cycling protocol: denaturation for 1 min at 95 °C followed by 40 cycles of 15 sec at 95 °C and 1 min at 60 °C. The primers are listed in Supplementary Table S3. A relative quantitative analysis was performed using the 2^−ΔΔ^Ct method.

## Additional Information

**How to cite this article**: Frattini, A. *et al.* High variability of genomic instability and gene expression profiling in different HeLa clones. *Sci. Rep.* **5**, 15377; doi: 10.1038/srep15377 (2015).

## Supplementary Material

Supplementary Information

## Figures and Tables

**Table 1:** HeLa cell line’s specific markers of HeLaSR and HeLaP lines.

Chromosome markers previously reported^17^,^18^,^19^,^20^	HeLaSR	HeLaP
M1 der(1)t(1;3)(q11;q11)	10/10	9/10
M2 der(1;9)(p11;q11)	0/10	4/10
M4 der(3;5)(p11;q11)	10/10	0/10
M5 der(3;20)(q10;q?10)	8/10	8/10
M7 i(5)(p10)	10/10	10/10
M8 der(7)t(7;19)(q35;?)	0/10	9/10
M10 der(9)t(3;9)(p21;p11)	0/10	8/10
M11 der(11)t(9;11;9)(?;p14?q33?;?dup(11)(p?)dup(11)(q?)	0/10	10/10
M14 der(19)t(13;19)(q21;p13)	10/10	10/10
M22 der (5;9)(p10;p10)	0/10	10/10
Loss or acquired chromosomes		
+chr.1	9/10	5/10
− chr. 3	10/10	10/10
− 2 chrs. 5	0/10	10/10
+chr.9	2/10	1/10
− chr. 9	0/10	3/10
− chr. 13	10/10	1/10
− chr. 19	2/10	10/10
Specific new markers		
i(1p)	1/10	0/10
i(5q)	0/10	10/10
t(3p;13q)	10/10	1/10
t(6;19)	10/10	1/10

Marker identification by FISH using WCP (Whole Chromosome Painting) for chr.1 vs chr. 3; chr. 5 vs chr. 9; chr.13 vs chr.19 (see Supplementary Fig. S1). Ten metaphases for each HeLa clone were analysed.
