# Supplementary material for: High variability of genomic instability and gene expression profiling in different HeLa clones
Source: Sci Rep. 2015 Oct 20;5:15377. doi: 10.1038/srep15377 (PMC4613361; doi:10.1038/srep15377)
Supplement: Supplementary Information [file srep15377-s1.pdf]

Supplementary Information

High variability of genomic instability and gene expression profiling in different HeLa clones

Annalisa Frattini[1,2*], Marco Fabbri[2], Roberto Valli[2], Elena De Paoli[2], Giuseppe Montalbano[2], Laura Gribaldo[3],Francesco Pasquali[2] and Emanuela Maserati.[2]

[1]Institute of Genetic and Biomedical Research (IRGB), National Research Council (CNR), via Fantoli 15/16, 20090 Milan, Italy. [2]Department of Clinical and Experimental Medicine (DMCS), University of Insubria, via J.H. Dunant,15, 21100 Varese, Italy. [3]Institute for Health and Consumer Protection, European Commission, via Enrico Fermi 2749, 21027 Ispra (VA), Italy.

**Correspondence and requests for materials should be addressed to A.F.*
(annalisa.frattini@irgb.cnr.it)

Table S1: Karyotypic variability within HeLa cell lines.
Figure S1: FISH analysis of HeLa lines.
Table S2: Primer used for qRT-PCR to validate the microarray results.
Table S3: Validation of genes mis-regulated by hypoxia by qRT-PCR in the HeLaSR and HeLaP.

Table S1: Karyotypic variability within HeLa cell lines.
Adapted from: Rutledge[1]

HeLa Cell Line	Modal Chr. Number	Provenience	Reference
HeLa-S3 (CRL-7924)	68 (range: 54 - 79)	ATCC	2
HeLa (ATCC® CCL-2™)	82 (range: 70 - 164)	ATCC	2
HeLa *Kyoto*	65 (range: 62 - 68)	CLS Cell Lines Service GmbH	3
HeLa	67	Undefined	4
HeLa CCL2	78 (range: 76 - 80)	ATCC	5
HeLa-S3	68	Provided by colleagues	6
HeLa-20	112	Provided by colleagues	6
HeLa-80	84	Provided by colleagues	6
HeLa	74 (range: 69 - 77)	Undefined	7
HeLa	65 (range: 62 - 67)	Provided by colleagues	8
HeLa D98/AH-2	62 (range 58 - 65)	Undefined	9
HeLa	84 (range: 58 - 179)	ATCC	10
HeLa-S3	68 (range: 51 - 74)	ATCC	10
HeLa	84 (range: 58 - 179)	ATCC	11
HeLa	77	Provided by colleagues	12
HeLa	75 (range: 73 - 76)	Provided by colleagues	12
HeLa D98/AG	63	Provided by colleagues	12
HeLa	69 (range:60 - 80)	Provided by colleagues	13
HeLa	60	Provided by colleagues	14
HeLa	69	Provided by colleagues	15
HeLa	71	Provided by colleagues	16
HeLa D98/AtH2	60 (range 57 - 63)	ATCC	17
HeLa D98/AH-2 cells	62 (range 57 - 63)	ATCC	17
HeLa	69	Provided by colleagues	18
HeLa A-CCL 2	77 (range: 56 - >86)	ATCC	19
HeLa 229-CCL2.1	78 (range: 56 - 86)	ATCC	19
HeLa G	69 (range: 62 - 72)	Company	19
HeLa S3G	76 (range: 65 - 79)	Company	19
HeLa65	65 (range: 62 - 69)	Provided by colleagues	19
HeLa71	70 (range: 59 - 72)	Provided by colleagues	19
Hela75	74 (range: 61 - 77)	Provided by colleagues	19
HeLa	59 (range: 57 - 64)	Provided by colleagues	20
HeLa	51	Undefined	21
HeLa	69 (range: 67-70)	Provided by colleagues	22
HeLa	61 (range: 59-62)	Provided by colleagues	22
HeLa St1	77	Provided by colleagues	23
HeLa F8	70	Provided by colleagues	23
HeLa S1 and S3	78 (range:75 - 82)	Provided by colleagues	24

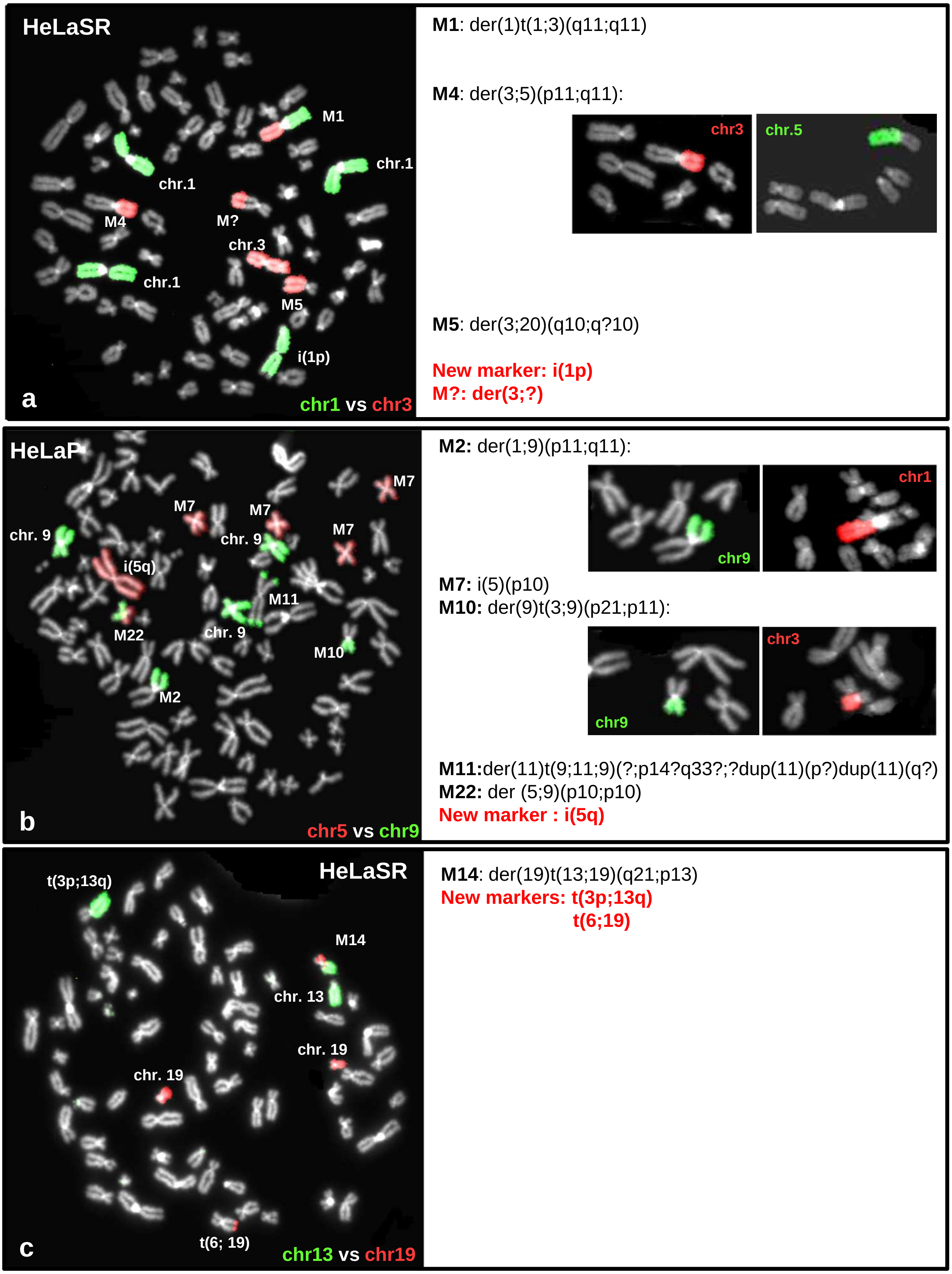

Figure S1: FISH analysis of HeLa lines. FISH analysis was performed using WCP (Whole Chromosome Painting) of chr.1 vs chr.3 (**a**); chr.5 vs chr.9 (**b**); chr.13 vs chr.19 (**c**). HeLa clones display almost all the HeLa-specific markers; in addition our FISH analysis highlights the appearance in each batch of new specific markers (see Table 1) (Leica D500B).

Table S2: Primer used for qRT-PCR to validate the microarray results.

Gene name	ac_number	Primer forward (5'→3')	Primer reverse (5'→3')
Adrenoceptor beta 2, surface	NM_000024.5	CAGGAAAGGGACGAGGTGTG	CAGCACATTGCCAAACACGA
BCL2-associated X protein	NM_001291428.1	AGCTGAGCGAGTGTCTCAAG	GGAAAAAGACCTCTCGGGGG
CCAAT/enhancer binding protein (C/EBP),beta	NM_001285878.1	GGCCGGTTTCGAAGTTGATG	GCTGACAGTTACACGTGGGT
Egl-9 family hypoxia-inducible factor 1	NM_022051.2	TTTTCTGGTCTGACCGTCGC	AGCTCGTGCTCTCTCATCTG
Egl-9 family hypoxia-inducible factor 3	NM_022073.3	AGCTTCCTCCTGTCCCTCAT	ATAGCAAGCCACCATTGCCT
Early growth response 1	NM_001964.2	CACCTGACCGCAGAGTCTTT	TTTGGCTGGGGTAACTGGTC
Heat shock protein 90kDa	NM_007355	TCTGGGTATCGGAAAGCAAGCC	GTGCACTTCCTCAGGCATCTTG
Hypoxia inducible lipid droplet-associated	NM_001098786.1	CGCTGGTGCTTAGTAACCGA	TTCTGAAAGGCCTCTGGACC
Immediate early response 3	NM_003897.3	CCGCAGGGTTCTCTACCCTC	AGAAGCCTTTTGGCTGGGTT
Ribosomal protein S18	NM_022551.2	TGTGGTGTTGAGGAAAGCA	CTTCAGTCGCTCCAGGTCTT
Ubiquitin C	NM_021009.6	GTGGCACAGCTAGTTCCGT	GTCAAGTGACGATCACAGCG
Tumour protein p53	NM_000546.5	AGAAAACCTACCAGGGCAGC	ACATCTTGTTGAGGGCAGGG
Vascular endothelial growth factor A isoform a	NM_001025366.2	TCACCAAGGCCAGCACATAG	TTTCTCCGCTCTGAGCAAGG

Table S3: Validation of genes regulated by hypoxia by qRT-PCR in the HeLaSR and HeLaP.

Detector		FC HeLaSR		FC HeLaP	
**Gene name**	**Gene symbol**	**qRT-PCR**	**microarray**	**qRT-PCR**	**microarray**
Adrenoceptor beta 2, surface	ADBR2	-3.2	-1.8	0.8	0.2
BCL2-associated X protein	BAX	-0.3	0.2	0.2	0.1
CCAAT/enhancer binding protein beta	CEBPB	1.8	1.4	0.4	0.1
Egl-9 family hypoxia-inducible factor 1	EGLN1	-1.6	1.5	4.8	1.4
Egl-9 family hypoxia-inducible factor 3	EGLN3	0.1	0.5	3.8	2.2
Early growth response 1	EGR1	-6.8	-6.3	1.7	1.0
Hypoxia inducible lipid droplet-associated	HILPDA	4.1	3.8	3.1	2.4
Immediate early response 3	IER3	6.4	2.8	2.2	1.1
Tumor protein p53	TP53	-0.4	-1.2	1.3	-0.3
Vascular endothelial growth factor A isoform a	VEGFA	1.3	4.3	4.9	2.2

Fold change (FC) expresses the difference of the mean log control and mean log hypoxia stimulated data.
FC qRT-PCR: regulation measured by real-time PCR technology; FC array: regulation done with microarray technology.

Supplementary references (related to Supplementary Table 1)

1. Rutledge S. What HeLa Cells Are You Using? (2014) Website https://thewinnower.com/papers/what-hela-cells-are-you-using Available at: insert website here. (Accessed: 19th May 2015).
2. ATCC Website http://www.lgcstandards-atcc.org/
3. Landry, J.J. *et al.* The genomic and transcriptomic landscape of a HeLa cell line. *G3* (Bethesda). **3,** 1213-1224 (2013).
4. Duesberg, P., Mandrioli, D., McCormack, A. & Nicholson, J.M. Is carcinogenesis a form of speciation. *Cell Cycle* **10**, 2100-2114 (2011).
5. Macville, M. *et al*. Comprehensive and definitive molecular cytogenetic characterization of HeLa cells by spectral karyotyping. *Cancer Res* **59,** 141-150 (1999).
6. Gille, J.J.P. & Joenje, H. Chromosomal instability and progressive loss of chromosomes in HeLa cells during adaptation to hyperoxic growth conditions. *Mutation Research/DNAging* **219,** 225-230 (1989).
7. Mincheva, A., Gissmann, L. & Zur Hausen, H. Chromosomal integration sites of human papillomavirus DNA in three cervical cancer cell lines mapped by in situ hybridization. *Medical Microbiology and Immunology* **176,** 245-256 (1987).
8. Ash, J.F., Fineman, R.M., Kalka, T., Morgan, M. & Wire, B. Amplification of sodium-and potassium-activated adenosinetriphosphatase in HeLa cells by ouabain step selection. *The Journal of Cell Biology* **99,** 971-983 (1984).
9. Stanbridge, E.J., Flandermeyer, R.R., Daniels, D.W & Nelson-Rees, W.A. Specific chromosome loss associated with the expression of tumorigenicity in human cell hybrids. *Somatic cell genetics* **7,** 699-712 (1981).
10. Lavappa, K. S. Survey of ATCC stocks of human cell lines for HeLa contamination. *In Vitro* **14,** 469–475 (1978).
11. Lavappa, K.S., Macy, M.L. & Shannon, J.E. Examination of ATCC stocks for HeLa marker chromosomes in human cell lines. *Nature* **259**, 211–213 (1976).
12. Heneen, W.K. HeLa cells and their possible contamination of other cell lines: karyotype studies. *Hereditas* **82,** 217-247 (1976).
13. Ghosh, S. & Ghosh, I. Variation of stemline karyotype in a HeLa cell line. *Zeitschrift für Krebsforschung und Klinische Onkologie* **84,** 129-133 (1975).
14. Obara, Y., Chai, L.S., Weinfeld, H. & Sandberg, A.A. Prophasing of interphase nuclei and induction of nuclear envelopes around metaphase chromosomes in HeLa and Chinese hamster homo-and heterokaryons. *The Journal of Cell Biology* **62,** 104-113 (1974).
15. Singer, R.M. & Fishman, W.H. Characterization of two HeLa sublines: TCRC-1 produces Regan isoenzyme and TCRC-2, non-Regan isoenzyme. *J Cell Biol* **60,** 777-780 (1974).

16. Czaker, R. Banding patterns and late replication in HeLa cells. *Humangenetik* **19,** 135-144 (1973).
17. Francke, U., Hammond, D.S. & Schneider, J.A. The band patterns of twelve D 98/AH-2 marker chromosomes and their use for identification of intraspecific cell hybrids. *Chromosoma* **41,** 111-121 (1973).
18. Ghosh, I. & Ghosh, S. Karyological studies on two HeLa lines. *Z Krebsforsch*. **74,** 103-9 (1970).
19. Bottomley, RH, Trainer, A.L. & Griffin, M.J. Enzymatic and chromosomal characterization of HeLa variants. *The Journal of Cell Biology* **41,** 806-815 (1969).
20. Dziekanowska, D. & Szurma, J. A hela subline with an established chromosome pattern and with a stemline chromosome number of 59. *Genetica* **39,** 237-244 (1968).
21. Hughes, D.T. The role of chromosomes in the characterisation of human neoplasms. *European Journal of Cancer* **1,** 233-243. (1965).
22. Cireli, E., Frimmel, J. & Schwarzacher, H.G. Cytogenetic studies on the HeLa cells. I. Chromosomes, DNA content and structure of the interphase nucleus. *Acta Anat* (Basel) **65,** 170-81 (1966).
23. Vogt, M. A. Study of the relationship between karyotype and phenotype in cloned lines of strain Hela. *Genetics* **44,** 1257 (1959).
24. Tjio, J.H. & Puck T.T. Genetics of somatic mammalian cells. II. Chromosomal constitution of cells in tissue culture. *J Exp Med*. **108,** 259-268 (1958).
